# Twenty‐Four‐Hour Blood Pressure‐Lowering Efficacy of Sacubitril/Valsartan Versus Olmesartan in Japanese Patients With Essential Hypertension Based on Nocturnal Blood Pressure Dipping Status: A Post Hoc Analysis of Data From a Randomized, Double‐Blind Multicenter Study

**DOI:** 10.1161/JAHA.122.027612

**Published:** 2023-04-07

**Authors:** Kazuomi Kario, Hiromi Rakugi, Daisuke Yarimizu, Yohei Morita, Shunsuke Eguchi, Kazuma Iekushi

**Affiliations:** ^1^ Division of Cardiovascular Medicine, Department of Medicine Jichi Medical University School of Medicine Tochigi Japan; ^2^ Department of Geriatric and General Medicine Osaka University Graduate School of Medicine Osaka Japan; ^3^ Medical Affairs Division Novartis Pharma K.K. Tokyo Japan

**Keywords:** circadian blood pressure pattern, hypertension, nocturnal blood pressure, olmesartan, sacubitril/valsartan, Hypertension, Treatment

## Abstract

**Background:**

Nighttime blood pressure (BP) and an abnormal nocturnal BP dipping profile are important cardiovascular risk factors in patients with hypertension. This post hoc analysis investigated the effects of sacubitril/valsartan on 24‐hour BP in patients with mild‐to‐moderate hypertension and in patient subgroups based on nocturnal BP dipping status.

**Methods and Results:**

Data from a randomized clinical trial comparing the BP‐lowering effects of 8 weeks of treatment with sacubitril/valsartan (200 or 400 mg/d) and olmesartan (20 mg/d) in Japanese patients with mild‐to‐moderate hypertension were analyzed. The primary end point was change in 24‐hour, daytime, and nighttime BP in patient subgroups based on nocturnal BP dipping status (dipper, nondipper). Six hundred thirty‐two patients with baseline and follow‐up ambulatory BP data were included. Both sacubitril/valsartan dosages reduced 24‐hour, daytime, and nighttime systolic BP, and 24‐hour and daytime diastolic BP, to a significantly greater extent than olmesartan in the dipper and nondipper groups. However, between‐group differences in nighttime systolic BP were more significant in the nondipper group (difference [95% CI] for sacubitril/valsartan 200 and 400 mg/d versus olmesartan 20 mg/d: –4.6 [95% CI, −7.3 to −1.8] and −6.8 [95% CI, −9.5 to −4.1] mm Hg, respectively; *P*<0.01 and *P*<0.001). Between‐group differences in the BP control rate were greatest in the nondipper subgroup (systolic BP control rate of 34.4% and 42.6% with sacubitril/valsartan 200 and 400 mg/d versus 23.1% with olmesartan 20 mg/d).

**Conclusions:**

This analysis highlights the value of sacubitril/valsartan therapy in patients with a nondipper profile of nocturnal BP and confirms this agent's potent 24‐hour BP‐lowering effect in Japanese populations with hypertension.

**Registration:**

URL: https://www.clinicaltrials.gov; Unique identifier: NCT01599104.

**Nonstandard Abbreviations and Acronyms**
DBP: diastolic blood pressure
SBP: systolic blood pressure

**Clinical Perspective**

**What Is New?**

- These are the first data on the differential 24‐hour blood pressure (BP)‐lowering effects of sacubitril/valsartan in patient subgroups based on the nocturnal BP dipping profile.
- Nondipper patients showed the most significant decreases in ambulatory BP (especially nighttime BP) during sacubitril/valsartan versus olmesartan treatment.

**What Are the Clinical Implications?**

- Given the contribution of nighttime BP and an abnormal nocturnal BP dipper pattern to cardiovascular risk, treatment with sacubitril/valsartan impacts multiple mechanisms that could improve cardiovascular outcomes in patients with hypertension.

A substantial body of evidence shows that nocturnal hypertension is a significant risk factor for developing cardiovascular disease and the occurrence of cardiovascular disease events.^1^, ^2^, ^3^, ^4^, ^5^, ^6^, ^7^, ^8^ In addition, changes in the normal circadian variation of blood pressure (BP) are also associated with increased cardiovascular risk.^9^, ^10^ Therefore, in addition to absolute BP values, attention to both nighttime BP and the circadian BP pattern is essential in mitigating cardiovascular risk in patients with hypertension.

Despite this, management of nocturnal hypertension remains an unmet medical need, because most current antihypertensive medication is given once daily in the morning. Unfortunately, this leaves a therapeutic blind spot in the early morning hours, where drug levels (and BP‐lowering effects) are at their lowest point before morning drug dosing. One approach to overcoming these issues is bedtime dosing of antihypertensive therapy.^11^, ^12^ There is also the possibility that novel agents may have favorable effects on circadian BP control in patients with hypertension.

One of these newer agents is the angiotensin receptor neprilysin inhibitor sacubitril/valsartan, which has recently been approved in Japan to treat hypertension.^13^ Studies in patients with hypertension have shown that sacubitril/valsartan significantly decreases office BP.^14^, ^15^, ^16^, ^17^, ^18^, ^19^, ^20^, ^21^, ^22^, ^23^, ^24^, ^25^, ^26^ More importantly, reductions in 24‐hour, daytime, and nighttime BP have been documented during treatment with sacubitril/valsartan in patients with hypertension.^14^, ^15^, ^17^, ^18^, ^20^, ^22^, ^23^, ^24^, ^25^ The latter effects are significant for cardiovascular risk reduction, but the impact of sacubitril/valsartan on ambulatory BP in patients with different circadian profiles of nighttime BP dipping (including the abnormal nondipper phenotype) has not yet been determined.

Therefore, this post hoc analysis of randomized clinical trial data evaluated the antihypertensive effects of sacubitril/valsartan compared with olmesartan on 24‐hour, daytime, and nighttime ambulatory BP in patients with mild‐to‐moderate hypertension and in patient subgroups (phenotypes) based on nocturnal BP dipping status.

## METHODS

### Study Design

The data that support the findings of this study are available from Novartis AG upon reasonable request.

This post hoc analysis used data from a multicenter, randomized, double‐blind, parallel‐group study comparing the BP‐lowering effects of sacubitril/valsartan and olmesartan in Japanese patients with mild‐to‐moderate essential hypertension (NCT01599104), conducted from June 2012 to April 2013.^26^ The study protocol was reviewed and approved by the institutional review board or independent ethics committee for each participating center. All study procedures were conducted following the *ICH Harmonized Tripartite Guidelines for Good Clinical Practice* (with applicable local regulations) and the ethical principles laid down in the Declaration of Helsinki. All patients provided written informed consent before initiation of all study procedures.

### Study Participants

Full details of patient inclusion and exclusion criteria have been reported previously.^26^ Briefly, eligible patients were ≥20 years of age and had treated or untreated mild‐to‐moderate systolic hypertension (mean seated systolic BP [SBP] ≥150 to <180 mm Hg); those with severe or secondary hypertension were excluded. Assuming that the reduction in 24‐hour ambulatory SBP would be 4 mm Hg greater in the sacubitril/valsartan group than in the olmesartan group (standard deviation, 12 mm Hg), it was calculated that the number of patients required to detect a between‐group with 90% power and a 2‐sided significance level of 5% was 573 (191 per group). Of the 1161 patients who participated in the randomized trial, those who agreed to have their ambulatory BP measured were eligible for inclusion in this analysis. In addition, nocturnal BP dipping status was defined as follows:

- Dipper: mean nighttime (10 pm–6 am) ambulatory BP fall of ≥10% versus daytime (6 am–10 pm) ambulatory BP
- Nondipper: mean nighttime ambulatory BP fall of <10% versus daytime ambulatory BP

### Study Procedures and Treatments

There was a 2‐ to 4‐week single‐blind, placebo run‐in period at baseline. The run‐in period for previously treated patients was 3 to 4 weeks, during which all existing antihypertensive therapy was tapered and discontinued, and the run‐in period for previously untreated patients was 2 weeks. After the run‐in period, patients meeting the entry criteria were randomized in a 1:1:1 ratio to 8 weeks' treatment with sacubitril/valsartan 200 mg/d, sacubitril/valsartan 400 mg/d, or olmesartan 20 mg/d, taken once daily in the morning.^26^ All adverse events were monitored during the study, and complete clinical laboratory testing was undertaken at screening, randomization, and the week 4 and week 8 follow‐up visits.

### Clinical End Points

The primary end point for this analysis was the change in 24‐hour, daytime, and nighttime BP from baseline to 8 weeks in patient subgroups based on nocturnal BP dipping status (dipper and nondipper). Secondary end points included the following: change from baseline to 8 weeks in 24‐hour BP; change from baseline to 8 weeks in levels of NT‐proBNP (N‐terminal pro‐B‐type natriuretic peptide); changes from baseline to 8 weeks in 24‐hour, daytime, and nighttime BP in patient subgroups based on baseline characteristics; rates of BP control at 8 weeks, where BP control was defined as 24‐hour SBP <130 mm Hg and 24‐hour diastolic BP (DBP) <80 mm Hg; safety in patient subgroups based on nocturnal BP dipping status; and between‐group differences in change from baseline to 8 weeks in the daytime and nighttime SBP.

### Follow‐Up and Assessments

Ambulatory BP measurement over 24 hours was conducted at 2 time points in a subset of participants in the original study. The first 24‐hour ambulatory BP measurement was performed after a patient had qualified for randomization based on office BP measurement but before administering the first dose of double‐blind study medication. The second ambulatory BP measurement was performed at week 8. Patients who withdrew from the study before week 8 did not need to have ambulatory BP measurements performed. At each 24‐hour ambulatory BP assessment period, the measurement device was attached to the nondominant arm, and both BP and heart rate were measured at study‐specified intervals. Readings were downloaded from device memory and then analyzed by a central laboratory.

### Statistical Analysis

The analysis population included patients with ambulatory BP data at baseline and all follow‐up visits. Demographics and baseline characteristics are presented using descriptive statistics, including mean±standard deviation, minimum, first quartile, median, third quartile, and maximum values for continuous variables, and frequencies and percentages for categorical variables. Between‐group differences were determined using an independent *t* test for continuous variables and a χ^2^ test for categorical variables. In addition, summaries with estimates and corresponding 95% CIs are presented, as appropriate. BP and laboratory baseline were defined as the measurements taken at the randomization visit. The follow‐up measurement of BP was obtained at 8 weeks.

ANCOVA was used to assess treatment effects on changes from baseline to 8 weeks in 24‐hour ambulatory BP in patient subgroups based on nocturnal BP dipping status. A repeated measures ANCOVA model with treatment, postdosing hours (time elapsed since drug administration), and treatment by postdosing hour interaction as fixed‐effect factors, and mean 24‐hour ambulatory SBP/DBP at baseline and 8 weeks as repeated measures was used. In addition, between‐group differences in mean change in 24‐hour ambulatory BP from baseline to 8 weeks with 95% CIs were determined.

Statistical analysis for secondary end points included 1‐way ANOVA, ANCOVA, least‐squares means with 95% CIs, and paired *t* test (see Data S1). If the overall treatment difference was significant in the model, the Tukey‐Kramer multiple comparison test was applied. Analyses were performed individually for each nocturnal BP dipping status group.

Statistical analyses were performed using SAS version 9.4 or higher. A 2‐sided *P* value of <0.05 was defined as statistically significant.

## RESULTS

### Study Population

Of the full analysis set from the randomized trial (n=1161), 632 patients (54% in total, 56% treated with sacubitril/valsartan 200 and 400 mg/d and 51% treated with olmesartan) had ambulatory BP data at baseline and follow‐up and were included in this analysis (Table, Table S1). Treatment groups were well matched at baseline (Table).

**Table 1:** Patient Demographics and Clinical Characteristics at Baseline, Overall, and by Treatment Group

Demographics and characteristics	Overall, n=632	Sacubitril/valsartan 200 mg/d, n=216	Sacubitril/valsartan 400 mg/d, n=216	Olmesartan 20 mg/d, n=200	*P* value
Age, y	58.7±10.8	57.8±11.1	58.5±10.7	59.9±10.5	0.145
Age ≥60 y, n (%)	304 (48.1)	96 (44.4)	104 (48.1)	104 (52.0)	0.305
Men, n (%)	439 (69.5)	149 (69.0)	150 (69.4)	140 (70.0)	0.975
BMI, kg/m^2^	25.6±3.7	25.4±3.6	25.5±3.8	25.8±3.8	0.670
BMI ≥25 kg/m^2^	324 (51.3)	111 (51.4)	108 (50.0)	105 (52.5)	0.948
Heart rate, beats/min	70.1±8.6	69.8±7.9	70.6±8.5	69.8±9.4	0.516
Heart rate ≥70 beats/min, n (%)	302 (47.8)	102 (47.2)	107 (49.5)	93 (46.5)	0.808
eGFR, mL/min per 1.73 m^2^	67.3±13.6	66.8±12.6	68.9±15.2	66.1±12.6	0.093
eGFR <60 mL/min per 1.73 m^2^, n (%)	199 (31.5)	59 (27.3)	67 (31.0)	73 (36.5)	0.129
NT‐proBNP, pg/mL*	66.8 (33.8–118.4)	65.1 (37.2–115.1)	67.3 (33.4–118.4)	67.7 (32.6–126.9)	0.834
NT‐proBNP ≥55 pg/mL, n (%)	239 (37.9)	81 (37.5)	81 (37.5)	77 (38.5)	0.842
NT‐proBNP ≥125 pg/mL, n (%)	92 (14.6)	29 (13.4)	29 (13.4)	34 (17.0)	0.760
Diabetes, n (%)	48 (7.6)	12 (5.6)	18 (8.3)	18 (9.0)	0.366
Dyslipidemia, n (%)	132 (20.9)	47 (21.8)	45 (20.8)	40 (20.0)	0.907
Antihypertensive drugs, n (%)†					
Calcium channel blocker	289 (45.7)	97 (44.9)	104 (48.1)	88 (44.0)	0.667
ACE inhibitor	20 (3.2)	5 (2.3)	7 (3.2)	8 (4.0)	0.616
ARB	296 (46.8)	99 (45.8)	95 (44.0)	102 (51.0)	0.335
β‐Blocker	28 (4.4)	10 (4.6)	7 (3.2)	11 (5.5)	0.527
Thiazide diuretic	17 (2.7)	5 (2.3)	4 (1.9)	8 (4.0)	0.367
Office BP					
SBP, mm Hg	158.0±6.9	157.5±6.5	158.9±7.4	157.5±6.8	0.071
DBP, mm Hg	93.8±9.8	93.6±9.6	94.3±10.1	93.5±9.8	0.673
PP, mm Hg	64.2±11.2	64.0±10.6	64.6±11.9	64.0±11.3	0.816
Ambulatory BP					
24‐hour SBP, mm Hg	148.0±11.7	147.8±11.7	148.7±11.0	147.3±12.3	0.447
24‐hour SBP ≥145 mm Hg, n (%)	374 (59.2)	127 (58.8)	134 (62.0)	113 (56.5)	0.512
24‐hour DBP, mm Hg	92.5±10.6	92.7±11.2	92.9±10.2	91.8±10.5	0.551
24‐hour DBP ≥90 mm Hg, n (%)	380 (60.1)	132 (61.1)	137 (63.4)	111 (55.5)	0.240
24‐hour PP, mm Hg	55.5±10.4	55.1±10.5	55.8±10.6	55.5±10.2	0.760
24‐hour PP ≥60 mm Hg, n (%)	194 (30.7)	62 (28.7)	70 (32.4)	62 (31.0)	0.701
Daytime SBP, mm Hg	152.1±12.0	151.9±11.8	152.8±11.5	151.6±12.9	0.555
Daytime DBP, mm Hg	95.7±11.1	95.9±11.7	96.1±10.7	95.0±10.8	0.612
Daytime PP, mm Hg	56.5±10.8	56.1±10.9	56.8±11.0	56.5±10.6	0.797
Nighttime SBP, mm Hg	139.5±13.6	139.3±13.7	140.5±12.7	138.8±14.4	0.412
Nighttime DBP, mm Hg	86.1±11.2	86.2±11.4	86.6±10.8	85.4±11.6	0.529
Nighttime PP, mm Hg	53.5±10.5	53.1±10.5	53.9±10.6	53.4±10.2	0.719
Nondipper, n (%)	382 (60.4)	125 (57.9)	136 (63.0)	121 (60.5)	0.632

Values are the number of patients (%) or mean±standard deviation. ACE indicates angiotensin‐converting enzyme; ARB, angiotensin receptor blocker; BMI, body mass index; BP, blood pressure; DBP, diastolic blood pressure; eGFR, estimated glomerular filtration rate; NT‐proBNP, N‐terminal pro‐B‐type natriuretic peptide; PP, pulse pressure; and SBP, systolic blood pressure.

* Data are available from 403 patients.

^**†**^ Three patients (1 in each treatment group) had treatment‐naïve hypertension.

### Overall Change in Ambulatory BP

**Figure 1:**
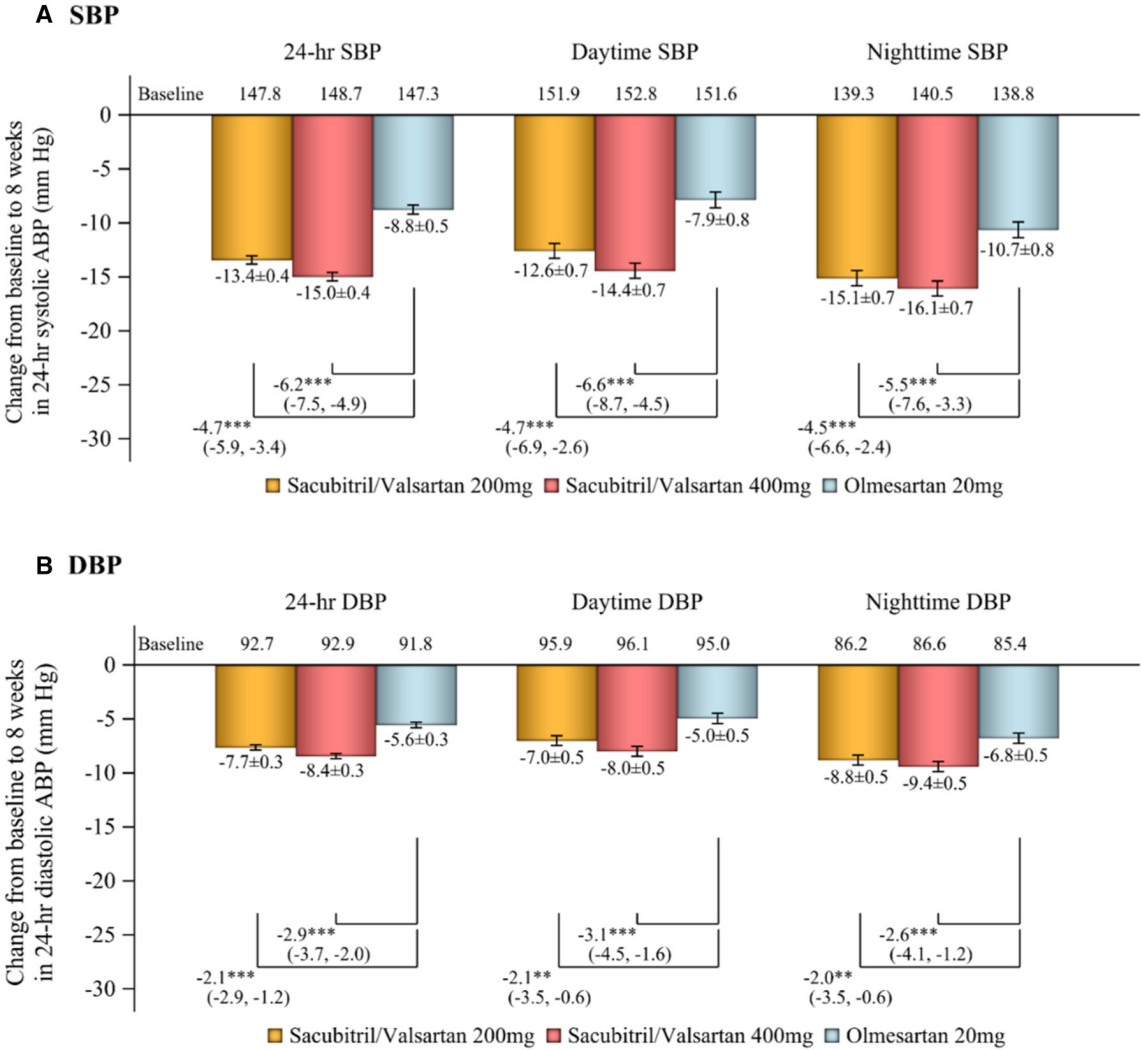
Change in ambulatory systolic blood pressure (A) and diastolic blood pressure (B) from baseline to week 8. Data are least‐squares mean change±standard error (repeated measures ANCOVA model). Daytime: 6 am to 10 pm; nighttime: 10 pm to 6 am. ABP indicates ambulatory blood pressure; DBP, diastolic blood pressure; and SBP, systolic blood pressure. *P<0.05, **P<0.01, ***P<0.001 vs olmesartan.

Changes in 24‐hour, daytime, and nighttime ambulatory SBP and DBP from baseline to week 8 were more significant with both dosages of sacubitril/valsartan compared with olmesartan (Figure 1). The proportion of patients with an ambulatory BP reduction of ≥5 mm Hg was 83% and 85% in the sacubitril/valsartan 200 and 400 mg/d groups, respectively, and 58% in the olmesartan group (Figure S1). The proportion of patients with an ambulatory BP reduction of ≥10 mm Hg was 63%, 69%, and 42%, respectively (Figure S1).

### Change in Ambulatory BP by Nocturnal Dipping Status

**Figure 2:**
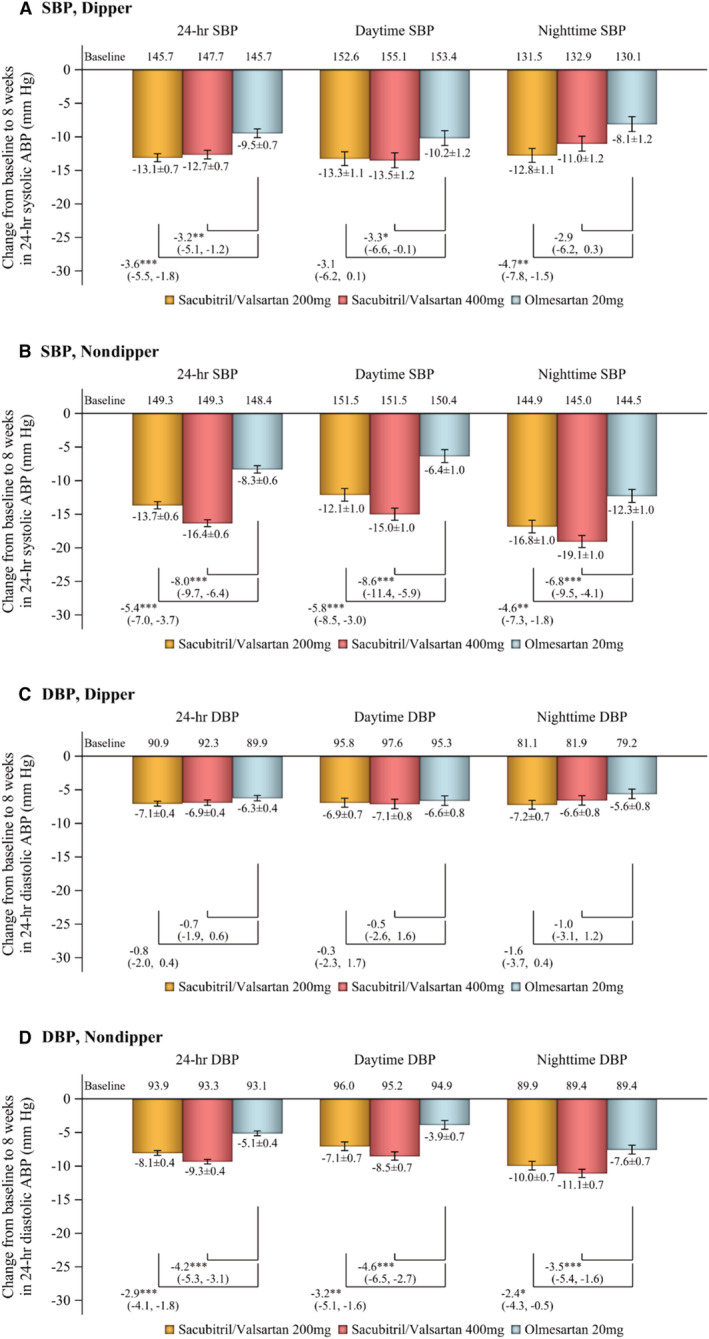
Change in ambulatory systolic blood pressure (A and B) and diastolic blood pressure (C and D) from baseline to week 8 in patient subgroups based on nocturnal blood pressure dipping status (dipper: A and C, nondipper: B and D). Data are least‐squares mean change±standard error (repeated‐measures ANCOVA model). Daytime: 6 am to 10 pm; nighttime, 10 pm to 6 am. ABP indicates ambulatory blood pressure; DBP, diastolic blood pressure; and SBP, systolic blood pressure. *P<0.05, **P<0.01, ***P<0.001 vs olmesartan.

Significant reductions in 24‐hour, daytime, and nighttime SBP and DBP from baseline to week 8 were seen during treatment with sacubitril/valsartan 200 and 400 mg/d and olmesartan 20 mg/d in both the dipper and nondipper groups (Figure 2). The magnitude of these reductions was greatest in the nondipper groups, where both dosages of sacubitril/valsartan reduced nighttime SBP and DBP to a significantly greater extent than olmesartan (Figure 2). Sacubitril/valsartan 200 and 400 mg/d decreased nighttime SBP (*P*=0.022 and *P*<0.001, respectively) and nighttime DBP (*P*=0.041 and *P*<0.001, respectively) to a significantly greater extent in nondippers compared with dippers (Figure S2). The reduction in 24‐hour SBP in sacubitril/valsartan 400 mg/d was greater in nondippers (−16.4 mm Hg) than in dippers (−12.7 mm Hg), with a between‐group difference of −3.7 mm Hg (*P*=0.004; Figure S2).

Sacubitril/valsartan was significantly more effective than olmesartan at lowering 24‐hour ambulatory SBP in all patient subgroups, except for those with diabetes (Figure S3). In addition, reductions in ambulatory BP during treatment with sacubitril/valsartan 200 mg/d were greater in women versus men and patients with a body mass index ≥25 versus <25 kg/m^2^ (Figure S3). For the 400 mg/d dosage of sacubitril/valsartan, ambulatory BP reductions were significantly greater in patients with a nondipper versus dipper profile and those with a baseline estimated glomerular filtration rate of <60 versus ≥60 mL/min per 1.73 m^2^, in men versus women and in patients with a body mass index <25 versus ≥25 kg/m^2^ (Figure S3).

**Figure 3:**
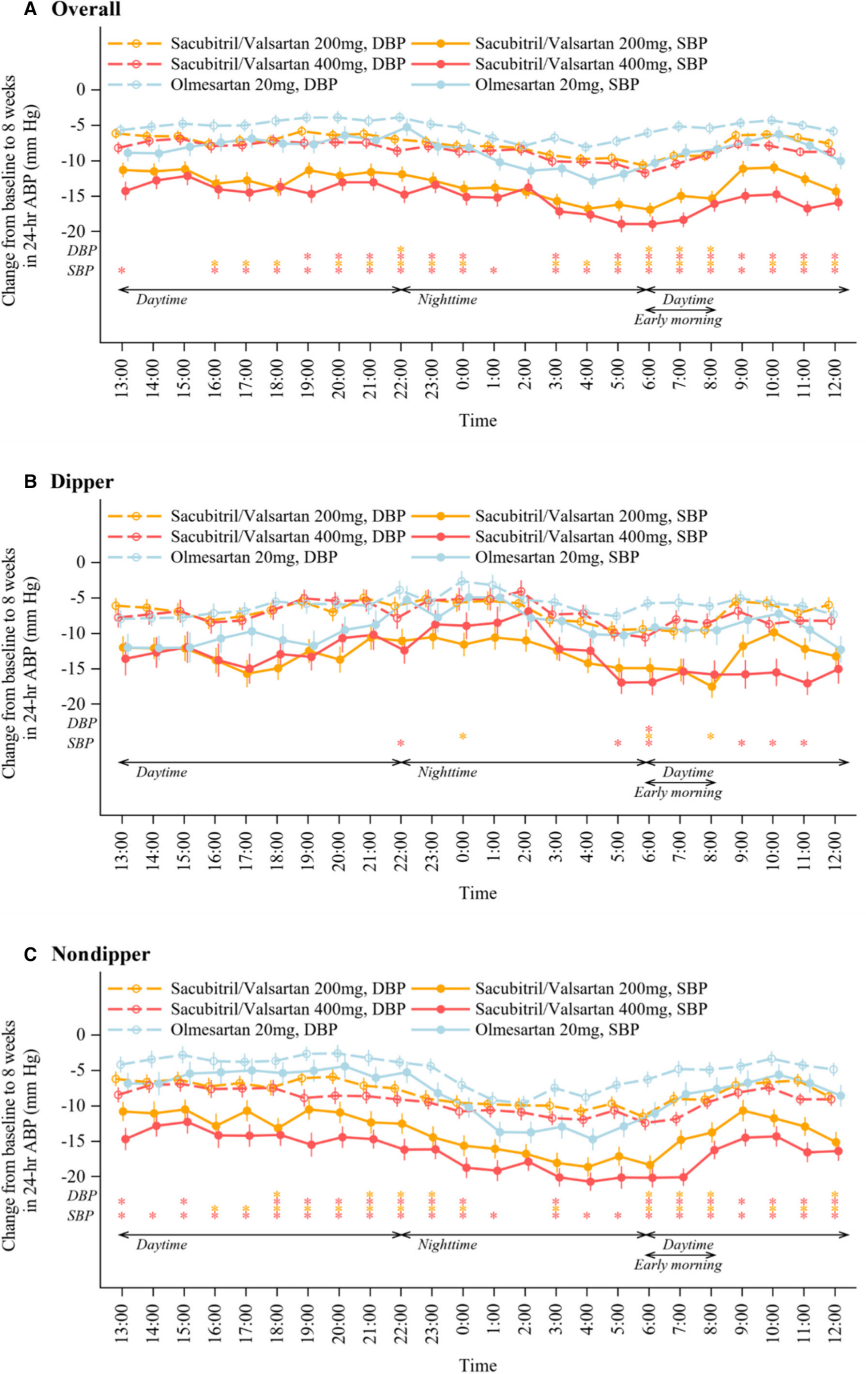
Time course of changes in 24‐hour systolic ambulatory blood pressure overall (A) and in patients with a dipper (B) or nondipper (C) profile of nocturnal blood pressure. ABP indicates ambulatory blood pressure; DBP, diastolic blood pressure; and SBP, systolic blood pressure. *P<0.05 vs olmesartan.

All treatments reduced SBP and DBP throughout the 24‐hour dosing interval (Figure 3). However, sacubitril/valsartan decreased SBP and DBP to the greatest extent in the early morning (from 6 am to 8 am), both overall and in patient subgroups based on dipper profile of nocturnal BP (Figure S4). These reductions in SBP and DBP were significantly greater with sacubitril/valsartan than with olmesartan (Figure S4). In addition, reductions in early morning ambulatory SBP and DBP with both sacubitril/valsartan and olmesartan were similar in the dipper and nondipper groups (Figure S5).

### Response to Therapy

**Figure 4:**
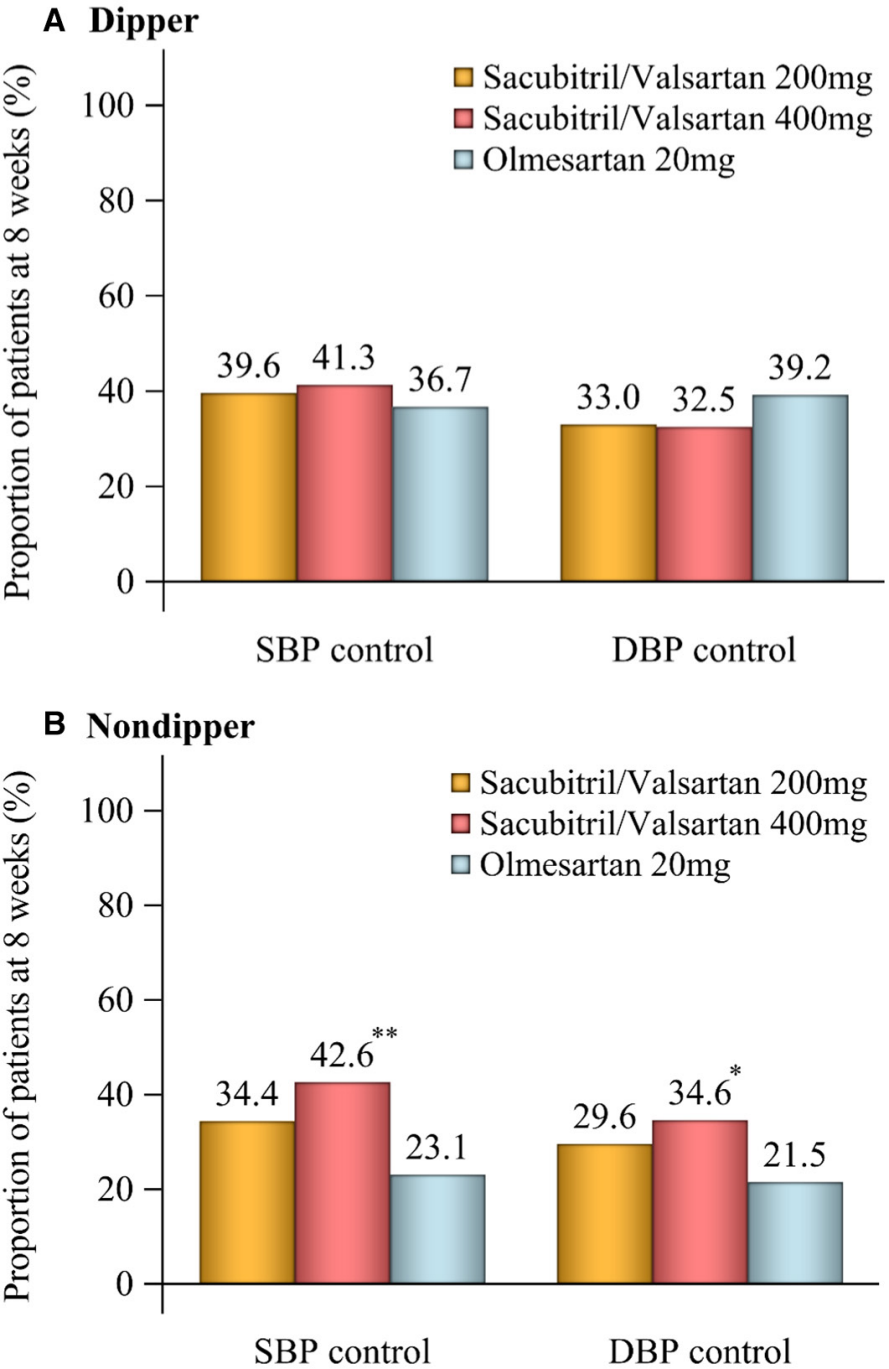
Blood pressure control rate at week 8 in patients with a dipper (A) or nondipper (B) profile of nocturnal blood pressure. SBP control: 24‐hour SBP <130 mm Hg. DBP control: 24‐hour DBP <80 mm Hg. DBP indicates diastolic blood pressure; and SBP, systolic blood pressure. *P<0.05, **P<0.001 vs olmesartan.

On average, >30% of all patients treated with sacubitril/valsartan were defined as responders at week 8 (ie, achieving BP control with 24‐hour SBP <130 mm Hg and 24‐hour DBP <80 mm Hg). In addition, BP control rates at week 8 were higher in both sacubitril/valsartan groups than with olmesartan in the nondipper subgroup, whereas differences between sacubitril/valsartan and olmesartan were less marked in the dipper subgroup (Figure 4). There was no difference between treatment groups in the proportion of patients who had a change in nocturnal BP dipping status during the study.

### Natriuretic Peptides

**Figure 5:**
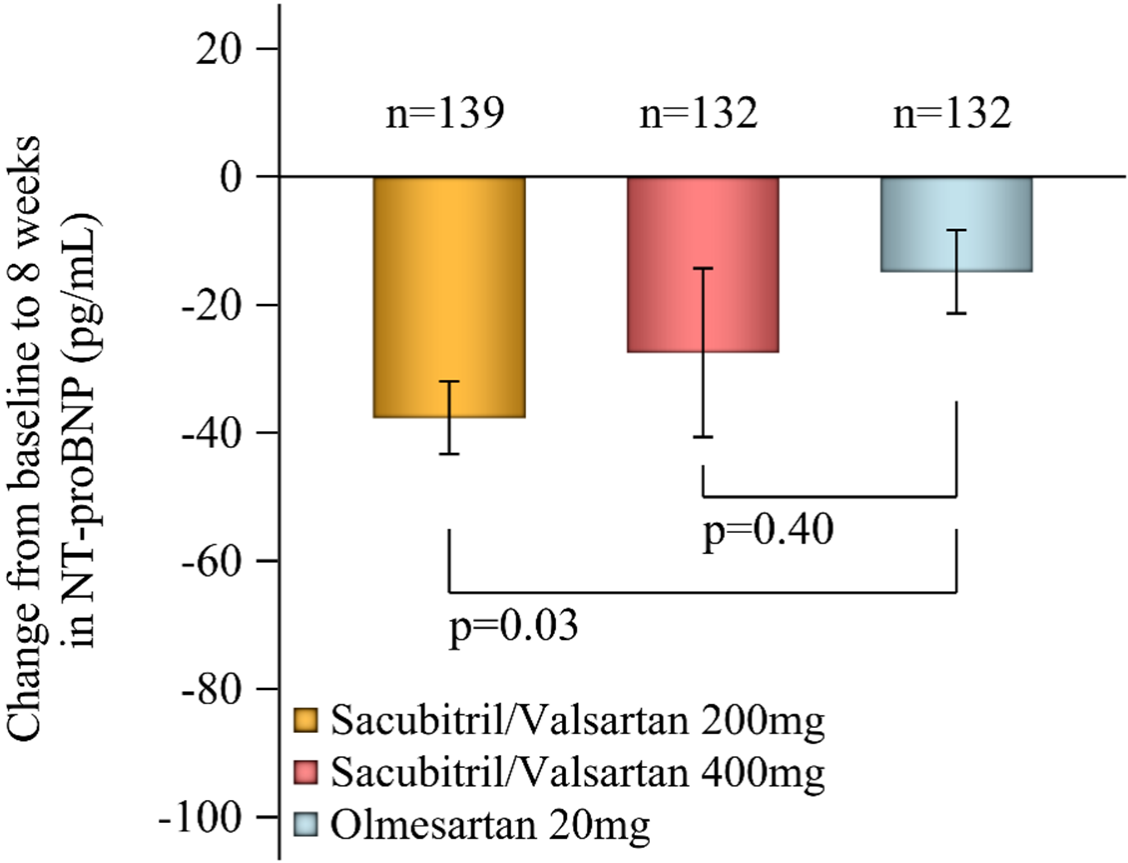
Overall change from baseline to week 8 in plasma NT‐proBNP levels (ANCOVA model; covariate: baseline NT‐proBNP). NT‐proBNP indicates N‐terminal pro‐B‐type natriuretic peptide.

NT‐proBNP levels were decreased to a greater extent in the sacubitril/valsartan groups compared with olmesartan, and the difference between sacubitril/valsartan 200 mg/d and olmesartan was statistically significant (Figure 5).

### Safety and Tolerability

All treatments were well tolerated, with no significant between‐group differences in reported adverse events (Tables S2 and S3).

## DISCUSSION

This analysis confirms the 24‐hour BP‐lowering effects of the angiotensin receptor neprilysin inhibitor sacubitril/valsartan in Japanese patients with mild‐to‐moderate hypertension and shows that these effects are greater than those of a midrange dose of the angiotensin receptor blocker olmesartan. Furthermore, we have also demonstrated that sacubitril/valsartan is an effective antihypertensive agent regardless of the nocturnal BP dipping profile and in various patient subgroups. Sacubitril/valsartan had similar antihypertensive effects on all ambulatory BP measures (24‐hour, daytime, and nighttime), although reductions in nighttime BP were slightly greater than the other 2 measures. This indicates consistent BP‐lowering effects throughout 24 hours with good antihypertensive coverage overnight and in the early morning predosing period.

In the current analysis, reductions from baseline in 24‐hour, daytime, and nighttime ambulatory SBP over 8 weeks of treatment with sacubitril/valsartan exceeded 12 mm Hg, and DBP reductions were all above 7 mm Hg (Figure 1). Given that a 5‐mm Hg reduction in SBP has been reported to reduce total cardiovascular disease risk by 10%, the risk of heart failure and stroke by 13% each, and the risk of coronary artery disease by 8%,^27^ the magnitude of BP reductions that occurred during treatment with sacubitril/valsartan in this study would be expected to have a positive impact on cardiovascular morbidity and mortality. Although olmesartan is considered to be one of the most potent angiotensin receptor blockers, the greater 24‐hour BP‐lowering effects with sacubitril/valsartan versus olmesartan are expected given that sacubitril/valsartan has a dual mechanism of action simultaneously inhibiting the angiotensin 2 type 1 receptor and neprilysin. Neprilysin inhibition increases circulating levels of natriuretic peptides, which promote natriuresis, diuresis, vasodilation, and endothelial permeability, and inhibit the renin‐angiotensin‐aldosterone system, sympathetic nervous system, aldosterone secretion, and fibrosis; these actions confer cardiac, vascular, and renal protection.^28^ Treatment with sacubitril/valsartan reduced NT‐proBNP levels compared with baseline and olmesartan in this study (Figure 5), which may help to reduce progression to heart failure by reducing cardiac load.^29^ The reason why the lower dosage of sacubitril/valsartan reduced NT‐proBNP to a greater extent than the higher dosage is not clear. However, it is possible that the higher dosage may cause slightly greater arterial dilation along with the obligatory small degree of volume expansion (a pseudotolerance effect), as seen with other nonspecific vasodilators, such as hydralazine.

Overall, the number of responders to treatment with sacubitril/valsartan was significantly higher than the number of responders to treatment with olmesartan, indicating the superior 24‐hour BP‐lowering efficacy of sacubitril/valsartan. Although a small proportion of patients had a change in 24‐hour BP of <5 mm Hg, most had reductions of ≥5 mm Hg, and some had a substantial response, with a ≥20 mm Hg reduction in 24‐hour BP during treatment (Figure S1). It would be interesting to compare these groups of patients to try and identify predictors of response to antihypertensive therapy with sacubitril/valsartan.

The novel finding of this study is the differences in the BP‐lowering effects of sacubitril/valsartan between patients with a dipper versus nondipper profile of nighttime BP (Figures 2 and 3). Although reductions in BP were clinically relevant in both patient groups, patients with a nonphysiological nondipper profile of nighttime BP showed the most significant decreases in ambulatory BP during treatment with sacubitril/valsartan. This was especially the case for nighttime BP and during the vulnerable period in the hours before waking. In addition, differences in the BP‐lowering effects of sacubitril/valsartan and olmesartan were much more evident in nondipper patients. Given that a nondipper profile of nighttime BP is an important cardiovascular risk factor,^9^, ^10^ this group of patients with hypertension might be especially suited to management with sacubitril/valsartan. For the 400 mg/d dosage of sacubitril/valsartan, ambulatory BP reductions were significantly greater in patients with a nondipper versus dipper profile (Figure S3B).

This study was conducted in Japanese patients, and there is evidence to suggest that angiotensin receptor neprilysin inhibitor drugs may be ideally suited for the treatment of hypertension in Asian populations.^30^ Asians are genetically more likely to have high salt sensitivity and have a higher level of salt consumption than Westerners.^31^, ^32^ Monotherapy with a renin‐angiotensin system inhibitor might be less effective in these individuals because the BP‐lowering effect of renin‐angiotensin system inhibitors is less in salt‐sensitive patients with a high salt intake, and renin‐angiotensin system inhibitors are less effective than calcium channel blockers for reducing 24‐hour BP in Asian patients.^33^ The change in neuropeptide levels that occurs during treatment with sacubitril/valsartan (as seen in this study) facilitates greater sodium excretion and may be one explanation for the greater reductions in BP (including nighttime BP) seen during treatment with sacubitril/valsartan in Asian patients compared with Westerners.^18^, ^20^ Effects on neuropeptides may also contribute to the normalization of the circadian rhythm of BP with sacubitril/valsartan.^34^

Although the data used were generated in a randomized controlled clinical trial, the post hoc analysis was not prespecified. The post hoc nature of this analysis means that potential sources of bias cannot be excluded. Therefore, the findings require confirmation in a prospective clinical study. In addition, only a single midrange dose of olmesartan was used as the comparator, and therefore both agents studied were not investigated across their full dosage range. Another limitation of the study was the single ethnicity population (Japanese), limiting the external generalizability of the findings.

## CONCLUSIONS

Sacubitril/valsartan has recently been approved in Japan and China for the treatment of hypertension. This analysis provides the first data on the differential effects of treatment with sacubitril/valsartan on ambulatory BP in patient subgroups based on the nocturnal BP dipping status. It also confirms the potent 24‐hour BP‐lowering effects of sacubitril/valsartan in Asian populations with hypertension. Strict reduction in 24‐hour BP and maintaining the dipper pattern of nocturnal BP are critical components of strategies designed to achieve perfect 24‐hour BP control, which is essential to attenuate or prevent hypertension‐mediated target organ damage and associated conditions.^35^ The effects of the angiotensin receptor neprilysin inhibitor sacubitril/valsartan on 24‐hour BP, especially when the nighttime pattern of BP dipping is abnormal, suggest that this agent might be ideally suited to control BP and reduce cardiovascular risk.

## Sources of Funding

This study was funded by Novartis.

## Disclosures

Dr Kario has received speaker's honoraria and consulting fees from Novartis Pharmaceuticals and Daiichi Sankyo, and a research grant from Daiichi Sankyo. In addition, Dr Rakugi served as the external medical expert for this study and has received consulting fees from Novartis Pharmaceuticals. D. Yarimizu, Dr Morita, S. Eguchi, and Dr Iekushi are employees of Novartis Pharma K.K., Tokyo, Japan.

## Supporting information

Data S1
Tables S1–S3
Figures S1–S5
