# Supplementary material for: Twenty‐Four‐Hour Blood Pressure‐Lowering Efficacy of Sacubitril/Valsartan Versus Olmesartan in Japanese Patients With Essential Hypertension Based on Nocturnal Blood Pressure Dipping Status: A Post Hoc Analysis of Data From a Randomized, Double‐Blind Multicenter Study
Source: J Am Heart Assoc. 2023 Apr 7;12(8):e027612. doi: 10.1161/JAHA.122.027612 (PMC10227270; doi:10.1161/JAHA.122.027612)
Supplement: Supplementary file 1 — Data S1 Tables S1–S3 Figures S1–S5 [file JAH3-12-e027612-s001.pdf]

SUPPLEMENTAL MATERIAL

Data S1. Supplemental Methods

Statistical analysis for primary endpoint

Change in 24-hour, daytime, and nighttime BP from baseline to 8 weeks

Hourly post-dosing values for systolic blood pressure (SBP) and diastolic blood pressure (DBP) were obtained over 24 hours by calculating the average of all readings taken in each hour. A repeated measure analysis of covariance (ANCOVA) method was used to assess effects of treatment on the change from baseline to 8 weeks in hourly SBP/DBP (hour 1, 2, 3, ..., 24) in patient subgroups based on nocturnal BP dipping status (dipper, non-dipper). The change from baseline to 8 weeks in SBP/DBP for each post-dosing hour was calculated by comparing the value for a specific hour at baseline with the same hour at 8 weeks. In the repeated measure ANCOVA model, treatment, post-dosing hour (hour 1, 2, 3, ..., 24) and treatment by post-dosing hour interaction were fixed-effect factors, and baseline mean 24-hour SBP/DBP was the repeated measure.

Statistical analysis for secondary endpoints

Change from baseline to 8 weeks in 24-hour BP

Mean change in SBP/DBP from baseline to 8 weeks for each clock hour was defined as the average of 8 weeks of readings for SBP/DBP minus baseline SBP/DBP at the corresponding clock hour. One way analysis of variance (ANOVA) models for the comparison between treatments were performed for each clock hour/post-dosing hour. If the overall treatment difference is significant in the ANOVA model, multiple comparison based on Tukey-Kramer was applied. The analysis is presented for all patients and by nocturnal BP dipping status.

Change from baseline to 8 weeks in levels of N-terminal pro-B-type natriuretic peptide (NT-proBNP)

Change in NT-proBNP level from baseline to 8 weeks is summarized using geometric mean. A paired t-test was used to analyze the difference in mean NT-proBNP level between baseline and 8 weeks for each treatment group for all patients and by nocturnal BP dipping status. Between-treatment differences in the change in NT-proBNP from baseline to 8 weeks was evaluated using an ANCOVA model with treatment as the fixed-effect and baseline NT-proBNP level as the covariate.

Changes from baseline to 8 weeks in 24-hour, daytime and nighttime BP in patient subgroups based on baseline characteristics

Data were analyzed using a repeated measure factorial ANCOVA model with treatment, baseline factor, post-dosing hours (hour 1, 2, 3, ..., 24), treatment by post-dosing hour interaction and treatment by baseline factor interaction as fixed-effect factors, and baseline mean 24-hour SBP as the repeated measure.

BP control rate at 8 weeks

The proportion of patients achieving control of 24-h ambulatory, daytime, and nighttime ambulatory SBP/DBP after 8 weeks is presented for the three treatment groups, and in patient subgroups based on nocturnal BP dipping status. An equality test was used to compare the proportion of patients achieving BP control between the sacubitril/valsartan and olmesartan groups.

Safety in patient subgroups based on nocturnal BP dipping status

Assessment of safety (in the safety analysis set) is based on the frequency of adverse events (AEs) and serious adverse events (SAEs) that were suspected by the investigators to be related to study medications. The incidence of any AEs, AEs leading to permanent treatment

discontinuation, any SAEs, and SAEs leading to discontinuations are summarized using descriptive statistics, by treatment group and by nocturnal BP dipping status.

Between-group differences in change from baseline to 8 weeks in daytime and nighttime SBP

Hourly post-dosing values for SBP were obtained over 24 hours by calculating the average of all readings taken in each hour. A repeated measure analysis of covariance (ANCOVA) method was used to assess effects of treatment on the change from baseline to 8 weeks in hourly SBP/DBP (hour 1, 2, 3, ..., 24) in patient subgroups based on nocturnal BP dipping status (dipper, non-dipper). The change from baseline to 8 weeks in SBP/DBP for each post-dosing hour was calculated by comparing the value for a specific hour at baseline with the same hour at 8 weeks. In the repeated measure ANCOVA model, treatment, post-dosing hour (hour 1, 2, 3, ..., 24) and treatment by post-dosing hour interaction were fixed-effect factors, and baseline mean 24-hour SBP was the repeated measure.

Table S1. Patient demographic and clinical characteristics at baseline in patient subgroups based on nocturnal blood pressure dipping status

	Overall (n=632)	Dipper (n=250)	Non-dipper (n=382)	p-value
Age, years	58.7±10.8	59.1±10.5	58.5±11.0	0.482
Age ≥60 years, n (%)	304 (48.1)	118 (47.2)	186 (48.7)	0.714
Male, n (%)	439 (69.5)	183 (73.2)	256 (67.0)	0.099
BMI, kg/m^2	25.6±3.7	25.4±3.8	25.6±3.7	0.505
BMI ≥25 kg/m^2, n (%)	324 (51.3)	121 (48.4)	203 (53.1)	0.505
Heart rate, beats/min	70.1±8.6	70.8±8.8	69.6±8.4	0.095
Heart rate ≥70 beats/min, n (%)	302 (47.8)	126 (50.4)	176 (46.1)	0.287
eGFR, mL/min/1.73m^2	67.3±13.6	69.0±13.8	66.2±13.4	0.011
eGFR <60 mL/min/1.73m^2, n (%)	199 (31.5)	66 (26.4)	133 (34.8)	0.026
NT-proBNP, pg/mL*	66.8 (33.8-118.4)	64.3 (33.8-112.5)	68.5 (33.8-121.8)	0.430
NT-proBNP ≥55 pg/mL, n (%)	239 (37.9)	60 (24.0)	104 (27.2)	0.834
NT-proBNP ≥125 pg/mL, n (%)	92 (14.6)	29 (11.6)	63 (16.5)	0.575
Diabetes, n (%)	48 (7.6)	18 (7.2)	30 (7.9)	0.762
Dyslipidemia, n (%)	132 (20.9)	55 (22.0)	77 (20.2)	0.577
**Antihypertensive drugs, n (%)**[†]				
Calcium channel blocker	289 (45.7)	122 (48.8)	167 (43.7)	0.210
ACE inhibitor	20 (3.2)	9 (3.6)	11 (2.9)	0.613
ARB	296 (46.8)	118 (47.2)	178 (46.6)	0.882
β-blocker	28 (4.4)	10 (4.0)	18 (4.7)	0.671
Thiazide diuretic	17 (2.7)	6 (2.4)	11 (2.9)	0.716
**Office BP**				
SBP, mmHg	158.0±6.9	158.5±6.9	157.6±6.9	0.126
DBP, mmHg	93.8±9.8	93.8±10.1	93.8±9.7	0.975
PP, mmHg	64.2±11.2	64.7±11.6	63.8±11.0	0.333
**Ambulatory BP**				
24-hour SBP, mmHg	148.0±11.7	146.3±11.8	149.0±11.5	0.004
24-hour SBP ≥145 mmHg, n (%)	374 (59.2)	140 (50.6)	234 (61.3)	0.189
24-hour DBP, mmHg	92.5±10.6	91.1±10.8	93.4±10.4	0.006
24-hour DBP ≥90 mmHg, n (%)	380 (60.1)	138 (55.2)	242 (63.4)	0.041
24-hour PP, mmHg	55.5±10.4	55.3±10.0	55.6±10.7	0.692
24-hour PP ≥60 mmHg, n (%)	194 (30.7)	71 (28.4)	123 (32.2)	0.311
Daytime SBP, mmHg	152.1±12.0	153.7±12.3	151.1±11.8	0.009
Daytime DBP, mmHg	95.7±11.1	96.2±11.4	95.3±10.8	0.345
Daytime PP, mmHg	56.5±10.8	57.5±10.8	55.8±10.8	0.052
Nighttime SBP, mmHg	139.5±13.6	131.5±11.8	144.8±12.1	<0.001

Nighttime DBP, mmHg	86.1±11.2	80.7±10.3	89.6±10.4	<0.001
Nighttime PP, mmHg	53.5±10.5	50.8±9.0	55.2±11.0	<0.001

Values are number of patients (%) or mean ± standard deviation.

Abbreviations: ACE, angiotensin-converting enzyme; ARB, angiotensin receptor blocker; BMI, body mass index; BP, blood pressure; DBP, diastolic blood pressure; eGFR, estimated glomerular filtration rate; NT-proBNP, N-terminal pro B-type natriuretic peptide; PP, pulse pressure; SBP, systolic blood pressure;

*Data available from 403 patients. † Three patients (one in each treatment group) had treatment-naïve hypertension.

Table S2. Adverse events by treatment group

	Sacubitril/valsartan			Olmesartan 20 mg
	200 mg (n=234)	400 mg (n=236)	Overall (n=470)	(n=230)
**Any adverse events**	84 (35.9)	82 (34.7)	166 (35.3)	94 (40.9)
Adverse event discontinuations	4 (1.7)	5 (2.1)	9 (1.9)	6 (2.6)
Drug-related adverse event discontinuations	1 (0.4)	2 (0.8)	3 (0.6)	3 (1.3)
**Serious adverse events**	1 (0.4)	1 (0.4)	2 (0.4)	4 (1.7)
Serious adverse event discontinuations	1 (0.4)	1 (0.4)	2 (0.4)	1 (0.4)
**Common adverse events**				
Nasopharyngitis	28 (12.0)	29 (12.3)	57 (12.1)	23 (10.0)
Influenza	1 (0.4)	2 (0.8)	3 (0.6)	0
Back pain	0	2 (0.8)	2 (0.4)	1 (0.4)
Pharyngitis	4 (1.7)	2 (0.8)	6 (1.3)	1 (0.4)
Upper respiratory tract infection	1 (0.4)	4 (1.7)	5 (1.1)	2 (0.9)
Dermatitis contact	1 (0.4)	4 (1.7)	5 (1.1)	1 (0.4)
Headache	5 (2.1)	3 (1.3)	8 (1.7)	2 (0.9)
Blood creatine phosphokinase increased	2 (0.9)	2 (0.8)	4 (0.9)	3 (1.3)
Blood bilirubin increased	1 (0.4)	1 (0.4)	2 (0.4)	4 (1.7)
Dizziness	1 (0.4)	1 (0.4)	2 (0.4)	1 (0.4)
Cystitis	4 (1.7)	2 (0.8)	6 (1.3)	0
Diarrhea	2 (0.9)	0	2 (0.4)	4 (1.7)
Alanine aminotransferase increased	0	0	0	3 (1.3)
Hypertension	0	0	0	3 (1.3)
Hepatic function abnormal	0	0	0	4 (1.7)

Table S3. Adverse events by treatment group in patient subgroups based on nocturnal blood pressure dipping status

	**Sacubitril/valsartan 200 mg/day**		**Sacubitril/valsartan 400 mg/day**		**Olmesartan 20 mg/day**	
	**Dipper (n=101)**	**Non-dipper (n=133)**	**Dipper (n=88)**	**Non-dipper (n=148)**	**Dipper (n=92)**	**Non-dipper (n=138)**
**Any adverse events**	36 (35.6)	48 (36.1)	27 (30.7)	55 (37.2)	36 (39.1)	58 (42.0)
AE discontinuations	1 (1.0)	3 (2.3)	0	5 (3.4)	1 (1.1)	5 (3.6)
Drug-related AE discontinuations	0	1 (0.8)	0	2 (1.4)	1 (1.1)	2 (1.4)
**Serious adverse events**	0	1 (0.8)	0	1 (0.7)	2 (2.2)	2 (1.4)
SAE discontinuations	0	1 (0.8)	0	1 (0.7)	0	1 (0.7)
**Common adverse events**						
Nasopharyngitis	11 (10.9)	17 (12.8)	8 (9.1)	21 (14.2)	9 (9.8)	14 (10.1)
Influenza	0	1 (0.8)	1 (1.1)	1 (0.7)	0	0
Back pain	0	0	1 (1.1)	1 (0.7)	1 (1.1)	0
Pharyngitis	2 (2.0)	2 (1.5)	1 (1.1)	1 (0.7)	0	1 (0.7)
Upper respiratory tract infection	0	1 (0.8)	1 (1.1)	3 (2.0)	0	2 (1.4)
Contact dermatitis	0	1 (0.8)	2 (2.3)	2 (1.4)	0	1 (0.7)
Headache	3 (3.0)	2 (1.5)	1 (1.1)	2 (1.4)	0	2 (1.4)
Increased blood creatine phosphokinase	1 (1.0)	1 (0.8)	0	2 (1.4)	0	3 (2.2)
Increased blood bilirubin	0	1 (0.8)	1 (1.1)	0	3 (3.3)	1 (0.7)
Dizziness	0	1 (0.8)	0	1 (0.7)	1 (1.1)	0
Cystitis	2 (2.0)	2 (1.5)	2 (2.3)	0	0	0
Diarrhea	1 (1.0)	1 (0.8)	0	0	2 (2.2)	2 (1.4)
Increased alanine aminotransferase	0	0	0	0	1 (1.1)	2 (1.4)
Hypertension	0	0	0	0	0	3 (2.2)
Hepatic dysfunction	0	0	0	0	1 (1.1)	3 (2.2)

Abbreviations: AE, adverse event; SAE, serious adverse event.

Figure S1. Individual changes in 24-hour ambulatory blood pressure from baseline to week 8 (waterfall plot) in the sacubitril/valsartan 200 mg/day (**A**), sacubitril/valsartan 400 mg/day (**B**) and olmesartan 20 mg/day (**C**) treatment groups.

A

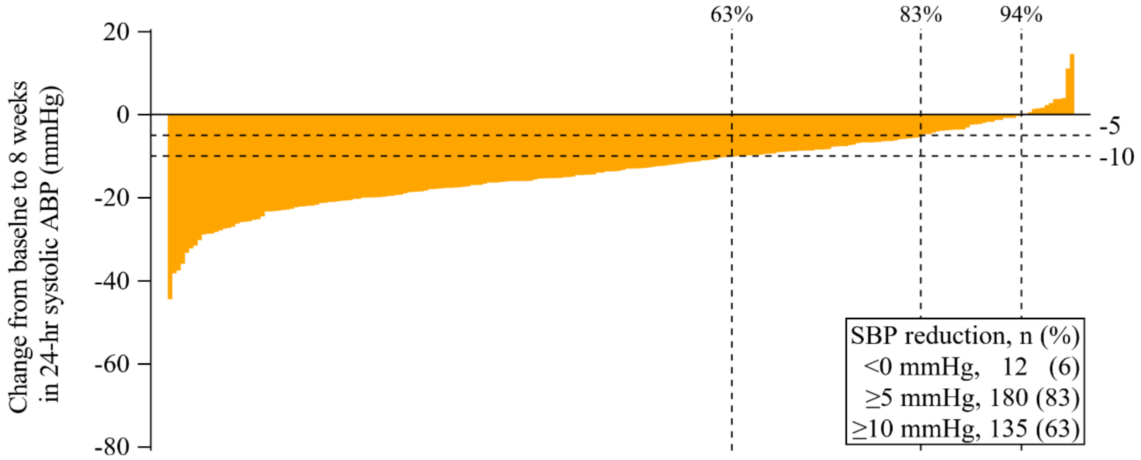

B

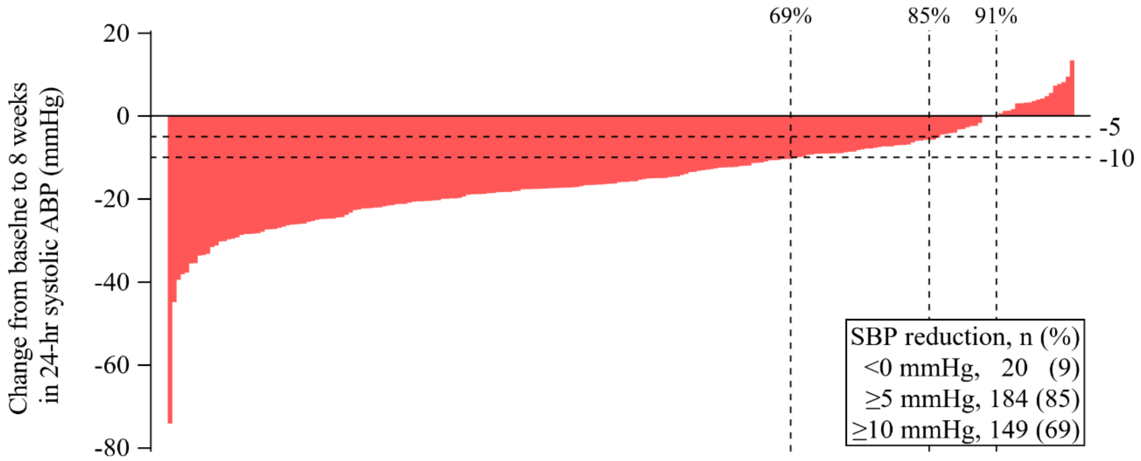

C

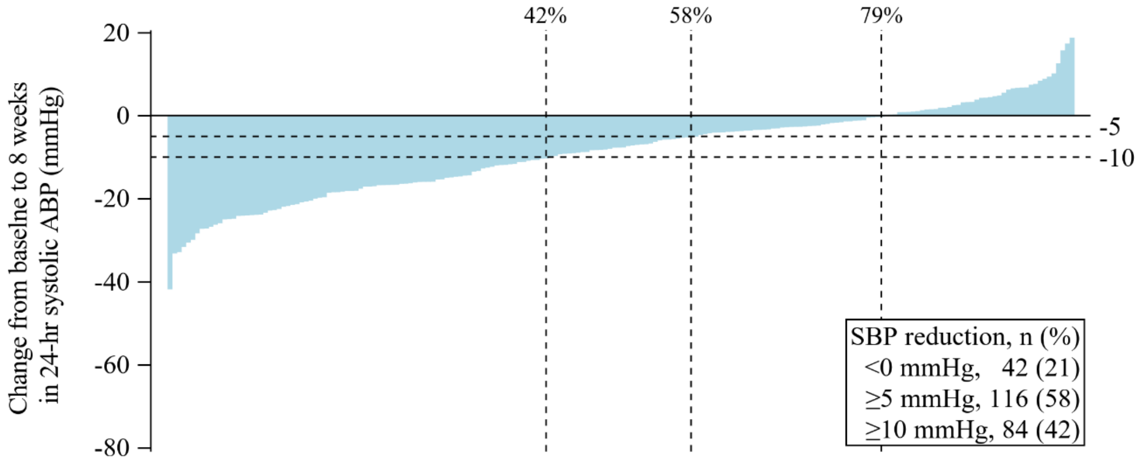

Figure S2. Change in ambulatory blood pressure from baseline to week 8 in patient subgroups based on nocturnal blood pressure dipping status, 24-hour SBP (**A**), daytime SBP (**B**), nighttime SBP (**C**), 24-hour DBP (**D**), daytime DBP (**E**), nighttime DBP (**F**). Data are least-squares mean change ± standard error (repeated-measures ANCOVA model).

A

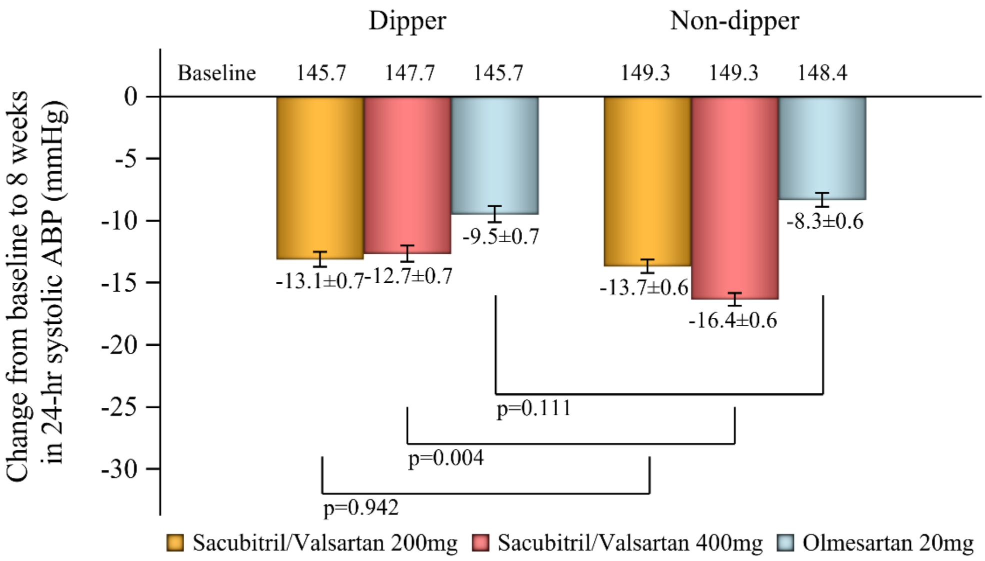

B

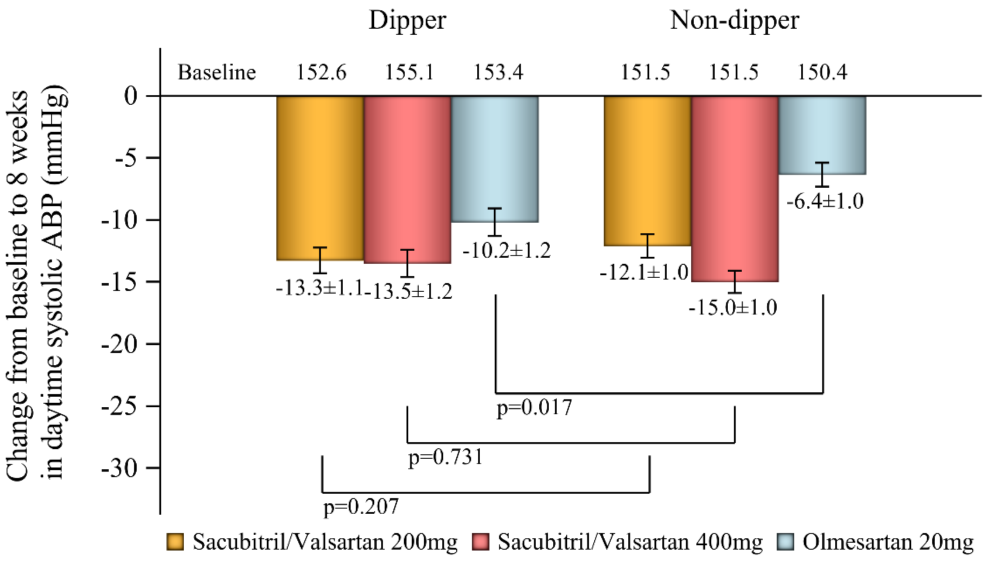

C

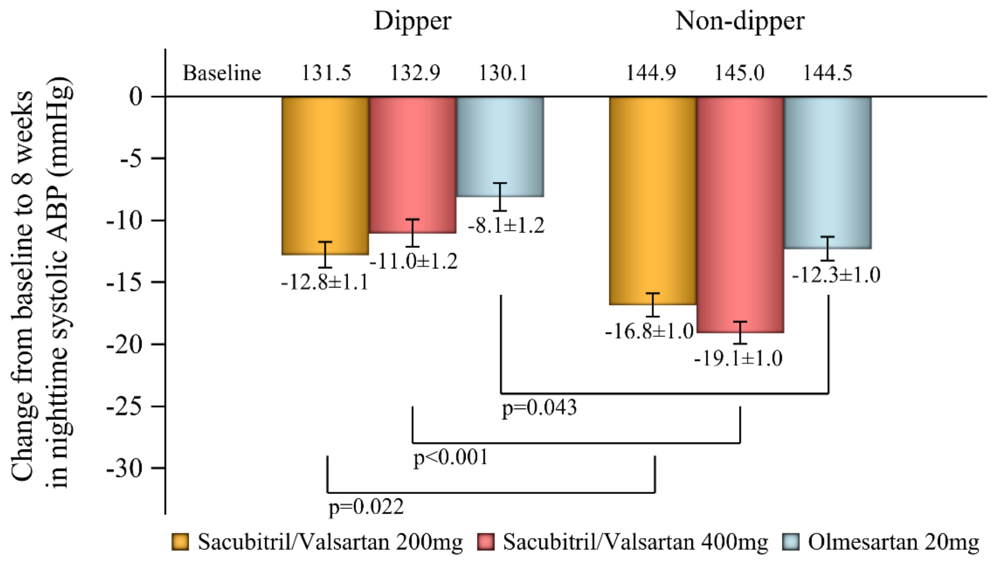
Dipper
Non-dipper
Baseline
131.5
132.9
130.1
144.9
145.0
144.5
-12.8±1.1
-11.0±1.2
-8.1±1.2
-16.8±1.0
-19.1±1.0
-12.3±1.0
p=0.043
p<0.001
p=0.022
Change from baseline to 8 weeks in nighttime systolic ABP (mmHg)
Sacubitril/Valsartan 200mg
Sacubitril/Valsartan 400mg
Olmesartan 20mg

D

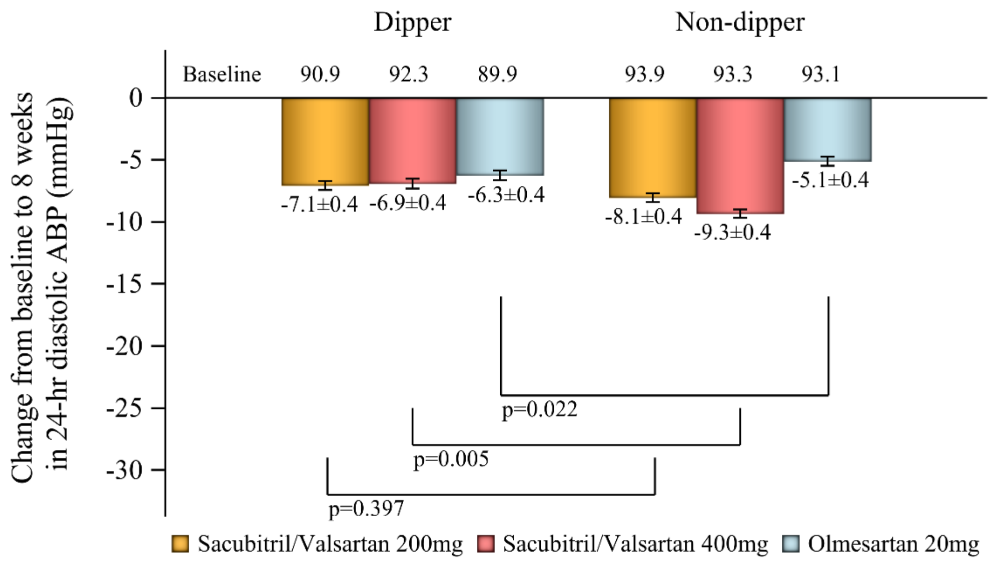
Dipper
Non-dipper
Baseline
90.9
92.3
89.9
93.9
93.3
93.1
-7.1±0.4
-6.9±0.4
-6.3±0.4
-8.1±0.4
-9.3±0.4
-5.1±0.4
p=0.022
p=0.005
p=0.397
Change from baseline to 8 weeks in 24-hr diastolic ABP (mmHg)
Sacubitril/Valsartan 200mg
Sacubitril/Valsartan 400mg
Olmesartan 20mg

E

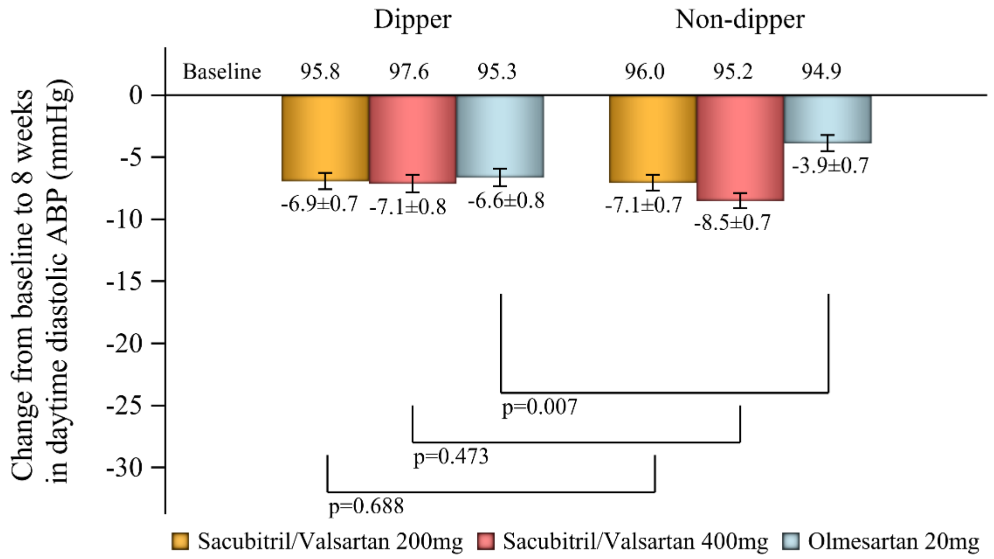

F

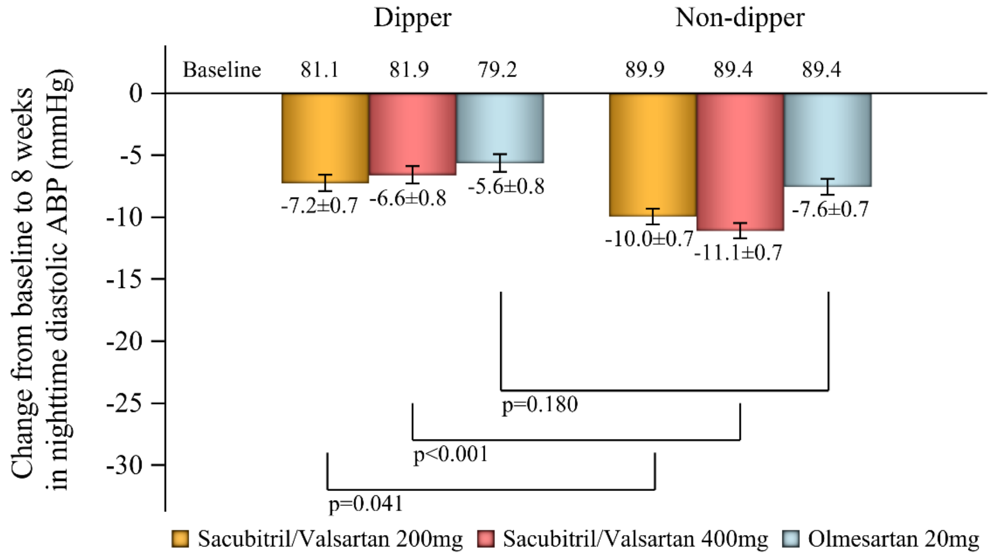

Abbreviations: ABP, ambulatory blood pressure; DBP, diastolic blood pressure; SBP, systolic blood pressure.

Figure S3. Differences in change in 24-hour ambulatory systolic blood pressure from baseline to week 8 during treatment with sacubitril valsartan 200 mg/day (**A**) or 400 mg/day (**B**) versus olmesartan 20 mg/day in patient subgroups based on baseline characteristics.

A

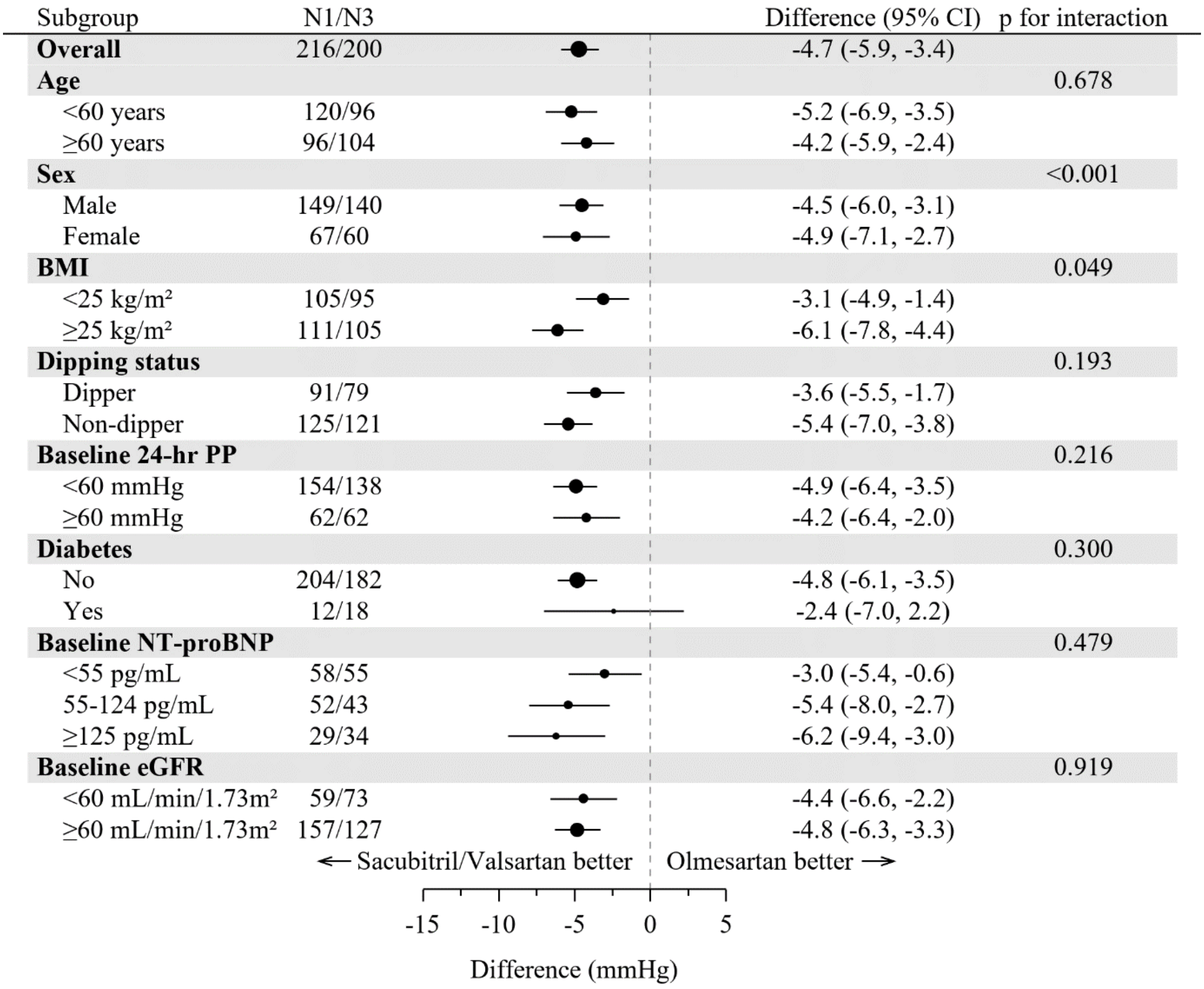

B

Subgroup	N2/N3	Difference (95% CI)	p for interaction
**Overall**	216/200	-6.2 (-7.5, -4.9)	
**Age**			0.531
<60 years	112/96	-6.9 (-8.8, -5.1)	
≥60 years	104/104	-5.5 (-7.3, -3.6)	
**Sex**			<0.001
Male	150/140	-6.7 (-8.3, -5.2)	
Female	66/60	-4.9 (-7.2, -2.5)	
**BMI**			0.003
<25 kg/m²	108/95	-6.7 (-8.6, -4.9)	
≥25 kg/m²	108/105	-5.5 (-7.4, -3.7)	
**Dipping status**			0.008
Dipper	80/79	-3.6 (-5.7, -1.5)	
Non-dipper	136/121	-7.8 (-9.4, -6.1)	
**Baseline 24-hr PP**			0.082
<60 mmHg	146/138	-5.8 (-7.4, -4.2)	
≥60 mmHg	70/62	-7.0 (-9.3, -4.7)	
**Diabetes**			0.817
No	198/182	-6.0 (-7.4, -4.7)	
Yes	18/18	-7.5 (-12.0, -3.1)	
**Baseline NT-proBNP**			0.424
<55 pg/mL	51/55	-5.3 (-7.9, -2.7)	
55-124 pg/mL	52/43	-6.1 (-8.9, -3.3)	
≥125 pg/mL	29/34	-6.2 (-9.6, -2.8)	
**Baseline eGFR**			0.006
<60 mL/min/1.73m²	67/73	-8.2 (-10.4, -5.9)	
≥60 mL/min/1.73m²	149/127	-5.3 (-6.9, -3.7)	

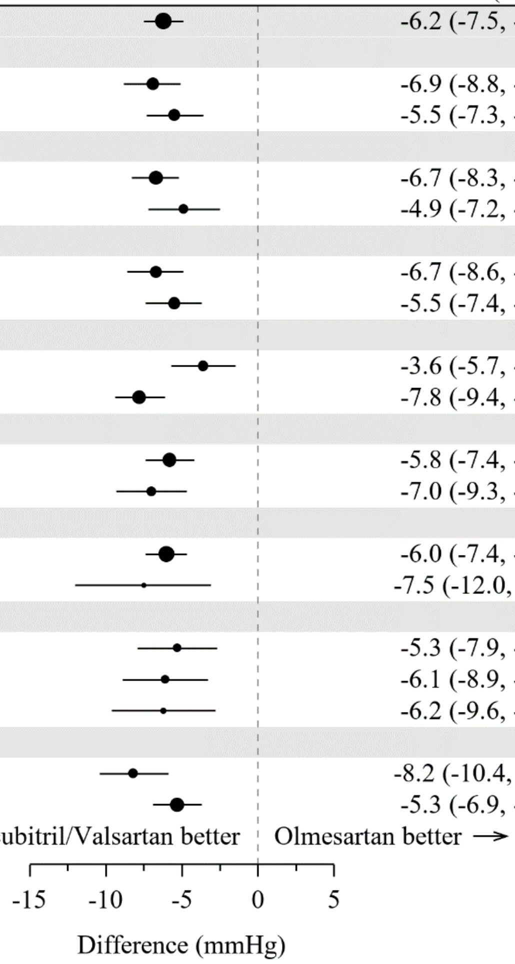

Abbreviations: BMI, body mass index; CI, confidence interval; eGFR, estimated glomerular filtration rate; NT-proBNP, N-terminal pro B-type natriuretic peptide; PP, pulse pressure; SBP, systolic blood pressure.

Figure S4. Change in early morning ambulatory systolic blood pressure (**A**) and diastolic blood pressure (**B**) from baseline to week 8 in the overall population and in patient subgroups based on nocturnal blood pressure dipping status (comparison between treatments). Data are least-squares mean change ± standard error (repeated-measures ANCOVA model). Early morning: 6am to 8am. *p<0.05, **p<0.01, ***p<0.001 vs. olmesartan.

A

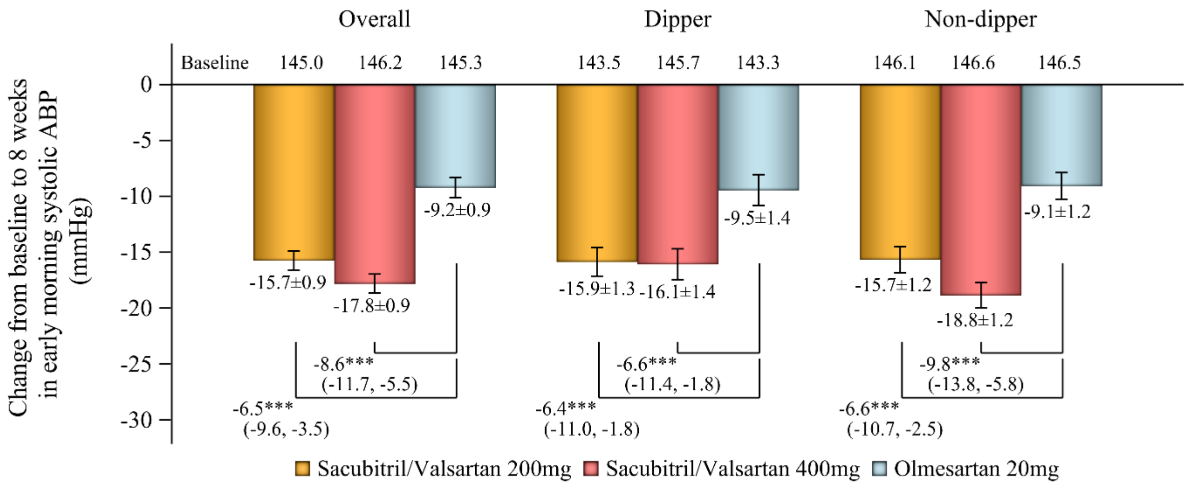

B

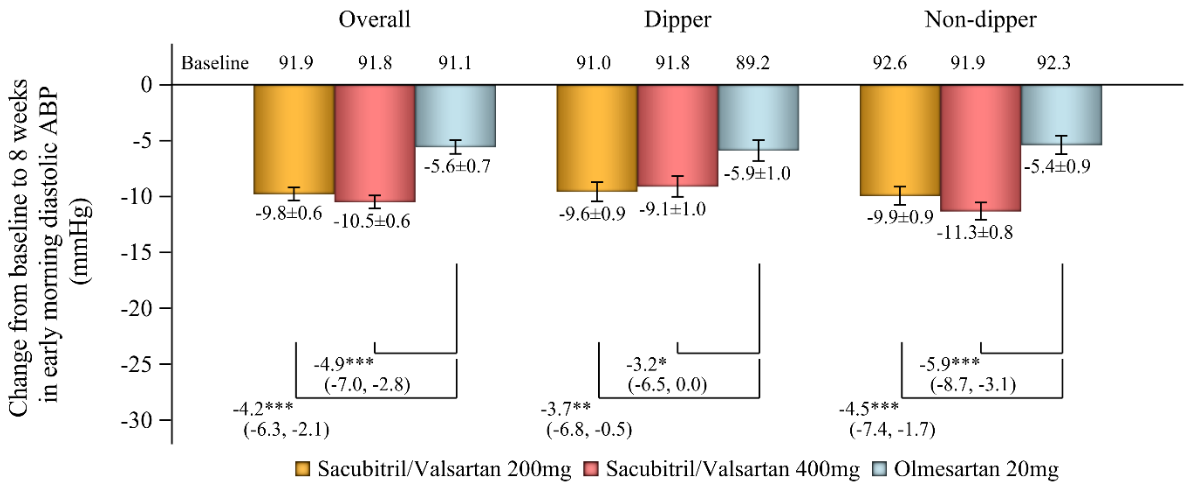

Abbreviations: ABP, ambulatory blood pressure; DBP, diastolic blood pressure; SBP, systolic blood pressure.

Figure S5. Change in early morning ambulatory systolic blood pressure (**A**) and diastolic blood pressure (**B**) from baseline to week 8 in patient subgroups based on nocturnal blood pressure dipping status (comparison between dipping status groups). Data are least-squares mean change ± standard error (repeated-measures ANCOVA model). Early morning: 6am to 8am.

A

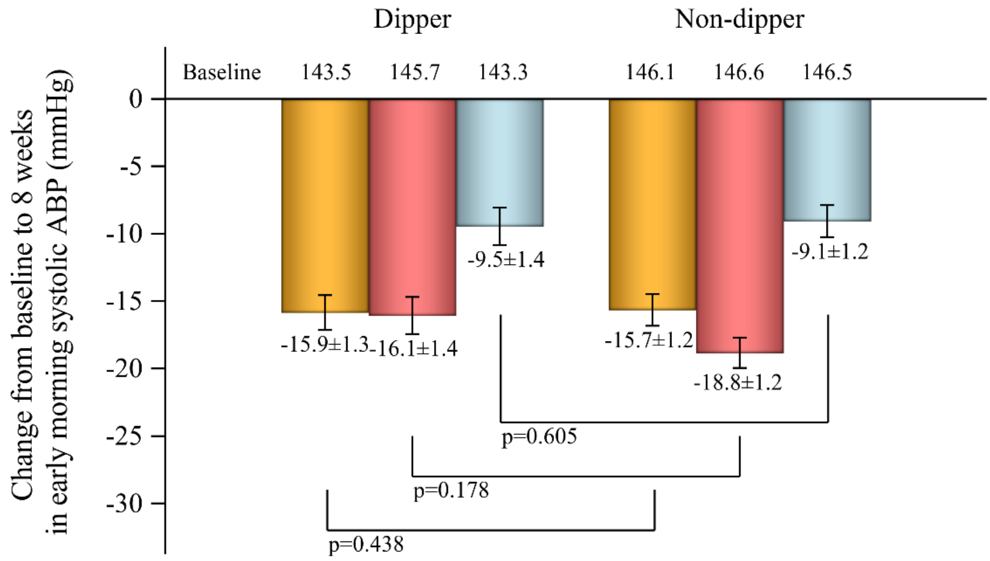

B

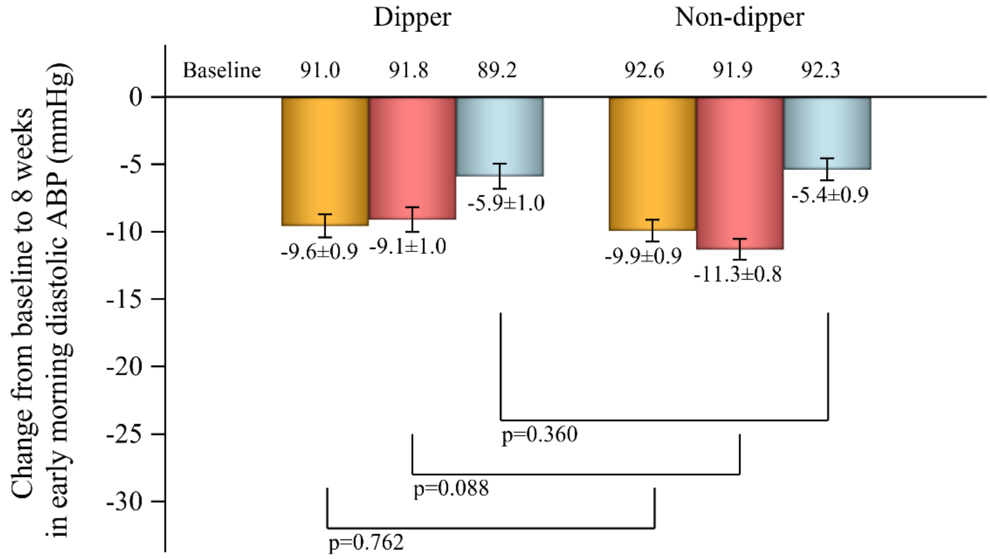

Abbreviations: ABP, ambulatory blood pressure; DBP, diastolic blood pressure; SBP, systolic blood pressure.
